# The Effect of COVID-19 Infection on Clinical Outcomes in Patients Undergoing Surgical Repair of Humerus Fractures

**DOI:** 10.7759/cureus.56780

**Published:** 2024-03-23

**Authors:** Nicholas M Scigliano, Troy B Puga, Noah M Scigliano, Yale K Williams, Michael A Boin

**Affiliations:** 1 Osteopathic Medicine, Kansas City University, Kansas, USA; 2 Medicine, University of Iowa, Iowa, USA; 3 Orthopedic Surgery Residency Program, HCA Research Medical Center, Kansas City University - Graduate Medical Education (GME) Consortium, Kansas, USA; 4 Orthopedic Surgery (Shoulder and Elbow), Orthopedic Health of Kansas City, Kansas, USA

**Keywords:** covid-19, epidemiology, humerus fractures, older adults, postoperative complications, orthopedic trauma during covid-19, risk factors

## Abstract

Introduction

Limited research exists on the association between coronavirus 2019 (COVID-19) infection and outcomes following surgical fixation for humerus fractures. The objective of this study was to evaluate the effects of COVID-19 on the clinical outcomes of patients undergoing humerus fracture surgery.

Methods

Approval to utilize insurance claim data from the Change Healthcare dataset was obtained from the Datavant COVID-19 Research Database. Patients older than 55 years old who underwent humerus fracture surgery from April 1, 2020, to March 1, 2022, were included in the analysis. COVID-19 status, comorbidities, and adverse events were identified using the International Classification of Diseases, 10th Revision (ICD-10) diagnostic codes. Propensity score matching with age, sex, and comorbidities was completed to create a 1:10 matched COVID-19-negative cohort. Univariate and multivariate logistic regressions were performed to assess the association of COVID-19 positivity with perioperative adverse events.

Results

A total of 18,365 patients underwent humerus fracture surgery in this study, of which 132 (0.72%) tested positive for COVID-19. Univariate analysis found that COVID-19-positive patients were at higher risk for myocardial infarction (5.30% vs. 1.74%, p = 0.015) and acute kidney injury (28.79% vs. 12.50%, p < 0.001) when compared to the 1:10 matched COVID-19-negative cohort. In addition, multivariate logistic regression found that COVID-19-positive patients had higher odds of experiencing any adverse event (2.57; 95% CI: 1.69-3.91; p < 0.001) or a minor adverse event (2.44; 95% CI: 1.57-3.79; p < 0.001).

Conclusion

COVID-19-positive patients have increased odds of experiencing adverse events after undergoing humerus fracture surgery in comparison to a matched COVID-19-negative control. Findings from this study stress the importance of using COVID-19 status as a factor in predicting outcomes following orthopedic surgery in this patient population.

## Introduction

From April 1, 2020, to March 1, 2022, the United States has 79 million confirmed cases of coronavirus 2019 (COVID-19) [1]. This two-year period profoundly impacted the field of orthopedics [2,3]. Cancellation of orthopedic surgical procedures led to a decrease in orthopedic patient admissions [4]. Upper extremity orthopedic patients were no exception as there were decreases in injury when compared to pre-pandemic numbers [5-7]. Since the pandemic's peak, there has been little investigation into the effects of COVID-19 on perioperative adverse events following surgical repair of humerus fractures.

Humerus fractures are responsible for 370,000 emergency room visits each year, with proximal humerus fractures constituting 50% of those cases [8]. In older adults, humerus fractures represent 1-3% of all fracture types [8]. The course of treatment for patients presenting with this type of injury can be nonoperative or operative. The types of operative treatment used for these patients include external fixation, intramedullary nailing, open reduction internal fixation, closed reduction percutaneous pinning, and arthroplasty [9]. In recent years, there has been an increase in the trend of using surgical fixation as a treatment for these fractures [10].

The timing from injury to surgery of humerus fractures is an important variable to consider in patients. The incidence of complications following surgical treatment of proximal humerus fractures significantly increases when surgery is delayed more than five days after the fracture event [11]. During the COVID-19 pandemic, postponement of surgery was frequently considered in older adults given their increased risk for morbidity and mortality when infected with COVID-19 [12,13]. These findings create a dilemma for orthopedic surgeons when conducting a risk assessment for COVID-19-positive patients indicated for humerus fracture surgery. Additionally, it demonstrates the need for literature analyzing the effects of COVID-19 infection on surgical fixation for humerus fractures.

The purpose of this study is to investigate comorbidities and adverse events of COVID-19-positive patients who underwent humerus fracture surgery over a two-year period. We hypothesize that COVID-19-positive patients will be at greater risk for adverse events following surgical repair of humerus fractures.

## Materials and methods

Data source

A retrospective cohort analysis was performed using the Change Healthcare dataset of the Datavant COVID-19 Research Database. Change Healthcare serves more than 1,000,000 providers; 5,500 hospitals; 2,400 payers; 39,000 pharmacies; 125,000 dentists; and 700 labs in the US.

The use of the Change Healthcare dataset was requested from the Datavant COVID-19 Research Database with a hypothesis-driven research proposal. The proposal application was reviewed by Datavant’s Scientific Steering Committee, which occurred in five sequential stages: compliance, technical, privacy, scientific, and data source review. After approval from Datavant’s Scientific Steering Committee, access to the COVID-19 Research Database was granted. Datasets are certified as Health Insurance Portability and Accountability Act compliant prior to being incorporated in the COVID-19 Research Database, and researchers are restricted access to the requested datasets. This study was deemed exempt from further review by our home Institutional Review Board (IRB) after completion of an IRB review exemption application.

Patient population

The Change Healthcare dataset was queried for claims received between April 1, 2020, and March 1, 2022. Patients who were born before the year 1966 were included in the study. Specifically, 1966 was used as a cutoff point to include only patients 55 years and older. The dataset was then queried for patients with a principal diagnosis of a humerus fracture using the International Classification of Diseases, 10th Revision (ICD-10) diagnostic codes. ICD-10 codes used to identify the principal diagnosis of a humerus fracture included S42.2, S42.3, and S42.4. These ICD-10 codes were queried to only include initial encounters for humerus fractures.

To identify the surgical treatment of humerus fractures, the ICD-10 Procedural Coding System (ICD-10-PCS) codes were used. The principal procedure for each patient was queried for all surgical approaches in the shoulder joint, humeral head, and humeral shaft. Patients with an ICD-10-PCS code for an external approach to surgical repair of humerus fractures were excluded. All duplicate patient claims were removed. Patient COVID-19 status was identified using the COVID-19 ICD-10 code U07.1. It is important to note that some patients who had COVID-19 during this date range may not have been diagnosed and received the U07.1 ICD-10 code for COVID-19.

Patient demographics and comorbidities

Patient sex and age were extracted for use in this study. Age was defined as the difference between the year of birth and the year 2020. ICD-10 codes (Table 1) were used to define comorbidities, which included asthma, chronic kidney disease, chronic obstructive pulmonary disease, congestive heart failure, coronary artery disease, diabetes, hypertension, and obesity.

**Table 1:** International Classification of Diseases, 10th Revision (ICD-10) diagnostic codes used to define the examined comorbidities. Source: Ref. [14]

Comorbidity	ICD-10 Diagnostic Code
Asthma	J45
Chronic Kidney Disease	N18
Chronic Obstructive Pulmonary Disease	J44
Congestive Heart Failure	I50
Coronary Artery Disease	I25
Diabetes	E10, E11, E12, E13, E14
Hypertension	I10, I11, I12, I13, I15
Obesity	E66

Adverse events

Patients who had undergone surgical treatment for all types of humerus fractures were assessed for adverse events using ICD-10 codes (Table 2). Adverse events were categorized as any adverse events, serious adverse events, and minor adverse events. Serious adverse events included cardiac arrest, deep venous thrombosis, myocardial infarction, pancreatitis, sepsis, surgical site infection, and venous thromboembolism. Minor adverse events included acute kidney injury, pneumonia, urinary tract infection, and wound dehiscence. Any adverse events included the occurrence of a minor adverse event, serious adverse event, or both a minor adverse event and a serious adverse event.

**Table 2:** ICD-10 diagnostic codes used to define the examined adverse events. Source: Ref. [14]

Adverse Event	ICD-10 Diagnostic Code
Surgical Site Infection	T8140, T8141, T8142, T8143, T8149
Sepsis	T8144, T8112, A41
Pulmonary Embolism	I26
Deep Venous Thrombosis	I82
Cardiac Arrest	I46, I9712
Myocardial Infarction	I21
Pancreatitis	K85
Pneumonia	J13, J14, J15, J16, J17, J18
Urinary Tract Infection	N39
Acute Kidney Injury	N17
Wound Dehiscence	T813

Data analysis

Patient claim data from the Change Healthcare dataset was stored and queried in a Snowflake data warehouse (Snowflake, San Mateo, CA). These data were accessed through a remote and secure Amazon Workspace (Amazon.com) desktop. All statistical analyses were performed in RStudio (RStudio Team, Boston, MA). Statistical significance for all tests in this study was set at an alpha level of 0.05.

A 1:10 COVID-19-negative cohort was created using propensity score matching. All COVID-19-positive patients were matched at a 1:10 ratio to COVID-19-negative patients who had similar propensity score values. Propensity scores were calculated using the age, sex, and comorbidities of the patients within each cohort. The matched 1:10 COVID-19-negative cohort was then used in comparison with the COVID-19-positive cohort to estimate the effect of COVID-19 exposure on adverse events following humerus fracture surgery. Propensity score matching was performed using the MatchIt (MatchIt: Nonparametric Preprocessing Causal Inference) computing package.

Univariate analysis of patient demographics, comorbidities, and adverse events was performed using Pearson’s chi-squared test with Yates continuity correction. The incidence of these variables was compared for both the matched and unmatched cohorts. Multivariate analysis was then performed on both unmatched and matched cohorts using logistic regression to determine the effect of COVID-19 positivity on adverse events. To perform logistic regression, a generalized linear model was constructed using a binomial family distribution and logit link functions in RStudio. Covariates in the generalized linear model included all demographic and comorbidity variables. The output of the model was then used to calculate odds ratios and 95% confidence intervals.

## Results

Patient population

A total of 18,385 patients who underwent surgical repair of humerus fractures from April 1, 2020, to March 1, 2022, met the inclusion criteria for this study. Of the total, 132 patients (0.72%) tested positive for COVID-19.

Patient demographics and comorbidities

Demographic characteristics of the COVID-19-positive cohort were compared with both the matched and unmatched COVID-19-negative cohort. COVID-19-positive patients were more likely to be male (44.70% vs. 23.42%, p < 0.001) and be 55-64.9 years old (31.06% vs. 16.67%, p < 0.001) when compared with the unmatched COVID-19-negative cohort (Table 3). When comparing the same cohorts, the COVID-19-positive cohort was less likely to be female as well (55.30% vs. 76.58%, p < 0.001) (Table 3). No additional statistically significant differences were observed when comparing age and sex between the COVID-19-positive cohort and both COVID-19-negative cohorts.

**Table 3:** Demographic characteristics of patients by COVID-19 status. *1:10 matching of year of birth, sex, and all comorbidities

Total Patients = 18,385	COVID-19 (-)		COVID-19 (+)			10:1 Matched COVID-19 (-) Cohort*		
	Number	Percent	Number	Percent	p-value	Number	Percent	p-value
	18,253	99.28%	132	0.72%		1320	7.18%	
Age								
55-64.9	3042	16.67%	41	31.06%	1.75E-05	442	33.48%	0.6407
65-74.9	7023	38.48%	40	30.30%	0.06669	394	29.85%	0.9928
>75	8188	44.86%	51	38.64%	0.1788	484	36.67%	0.7243
Sex								
Male	4275	23.42%	59	44.70%	1.75E-08	591	44.77%	1
Female	13,978	76.58%	73	55.30%	1.75E-08	729	55.23%	1

Comorbidity characteristics of the COVID-19-positive cohort were also compared with both the matched and unmatched COVID-19-negative cohorts using a univariate analysis. COVID-19-positive patients were more likely to have congestive heart failure (25.76% vs. 9.30%, p < 0.001) when compared with the unmatched COVID-19-negative patients. COVID-19-positive patients were more likely to have asthma (0.00% vs. 0.00%, p < 0.001) and hypertension (48.48% vs. 37.58%, p = 0.01821) in comparison to the matched COVID-19-negative patients (Table 4). No additional statistically significant differences in comorbidity variables between the cohorts were observed.

**Table 4:** Comorbidity characteristics of patients by COVID-19 status. *1:10 matching of year of birth, sex, and all comorbidities

Total Patients = 18,385	COVID-19 (-)		COVID-19 (+)			10:1 Matched COVID-19 (-) Cohort*		
	Number	Percent	Number	Percent	p-value	Number	Percent	p-value
	18,253	99.28%	132	0.72%		1320	7.18%	
Asthma	593	3.25%	0	0.00%	0.06319	0	0.00%	<2.2e-16
Chronic Kidney Disease	1365	7.48%	11	8.33%	0.8368	82	6.21%	0.4457
Congestive Heart Failure	1698	9.30%	34	25.76%	3.00E-10	296	22.42%	0.4458
Coronary Artery Disease	1752	9.60%	18	13.64%	1.56E-01	177	13.40%	1
COPD	1528	8.37%	10	7.58%	0.8641	133	10.10%	0.4437
Diabetes	4036	22.11%	23	17.42%	0.2347	209	15.83%	0.7255
Hypertension	9726	53.28%	64	48.48%	0.3107	496	37.58%	0.01821
Obesity	1931	10.58%	7	5.30%	0.06806	111	8.41%	0.281

Adverse events

Adverse events occurring in all cohorts were compared using univariate and multivariate analyses (Table 5). In comparison to the unmatched COVID-19-negative cohort, a univariate analysis observed that COVID-19-positive patients had higher rates of any adverse events (37.12% vs. 19.04%, p < 0.001) and minor adverse events (32.58% vs. 17.36%, p <0.001). COVID-19-positive patients were also more likely to experience specific complications, including myocardial infarction (5.30% vs. 1.02%, p < 0.001), sepsis (3.03% vs. 0.59%, p = 0.0023), and acute kidney injury (28.79% vs. 11.40%, p < 0.001).

**Table 5:** Univariate analysis of adverse events by COVID-19 status. *1:10 matching of year of birth, sex, and all comorbidities

Total Patients = 18,385	COVID-19 (-)		COVID-19 (+)			10:1 Matched COVID-19 (-) Cohort*		
	Number	Percent	Number	Percent	p-value	Number	Percent	p-value
	18,253	99.28%	132	0.72%		1320	7.18%	
Any Adverse Event	3476	19.04%	49	37.12%	2.66E-07	281	21.29%	5.58E-05
Serious Adverse Event	520	2.85%	9	6.82%	0.01401	60	4.55%	0.3392
Cardiac Arrest	29	0.16%	0	0.00%	1	5	0.38%	1
Deep Venous Thromboembolism	137	0.75%	2	1.52%	0.6127	17	1.29%	1
Myocardial Infarction	187	1.02%	7	5.30%	1.27E-05	23	1.74%	0.01547
Pancreatitis	8	0.04%	0	0.00%	1	1	0.08%	1
Pulmonary Embolism	92	0.50%	0	0.00%	0.8425	7	0.53%	0.8574
Sepsis	107	0.59%	4	3.03%	0.002304	12	0.91%	0.07367
Surgical Site Infection	5	0.03%	0	0.00%	1	0	0.00%	<2.2e-16
Minor Adverse Event	3168	17.36%	43	32.58%	7.68E-06	241	18.26%	0.0001234
Acute Kidney Injury	2080	11.40%	38	28.79%	1.06E-09	165	12.50%	5.34E-07
Pneumonia	318	1.74%	0	0.00%	0.2322	34	2.58%	0.1178
Urinary Tract Infection	1092	5.98%	9	6.82%	0.8266	63	4.77%	0.4111
Wound Dehiscence	11	0.06%	0	0.00%	1	1	0.08%	1

COVID-19-positive patients maintained a statistically significant difference, demonstrating higher rates of any adverse events (37.12% vs. 21.29%, p < 0.001) and minor adverse events (32.58% vs. 18.26%, p < 0.001) when compared to the 1:10 propensity score matched COVID-19-negative cohort. COVID-19-positive patients were more likely to experience myocardial infarction (5.30% vs. 1.74%, p = 0.015) and acute kidney injury (28.79% vs. 12.50%, p < 0.001). Lastly, it was found that there was a statistically significant difference in rates of surgical site infection between the 1:10 propensity score matched COVID-19-negative cohort and the COVID-19-positive cohort (0.00% vs. 0.00%, p < 0.001).

**Figure 1:**
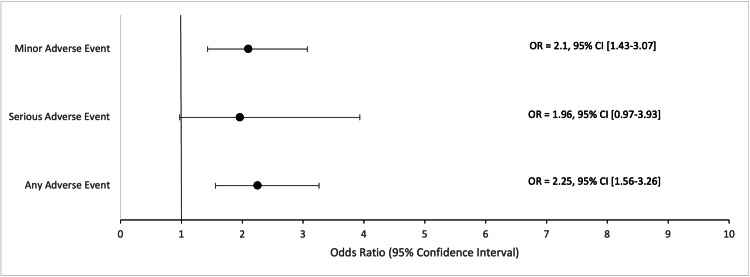
Unmatched multivariate odds ratios in COVID-19 (+) patients who underwent humerus fracture surgery. Forest plot displaying multivariate odds ratios and corresponding 95% confidence intervals for adverse events in COVID-19-positive patients who underwent humerus fracture surgery. The comparison group in the logistic regression model was the unmatched COVID-19-negative cohort. This model controlled for age, sex, and comorbidities. OR = Odds Ratio; CI = Confidence Interval

**Figure 2:**
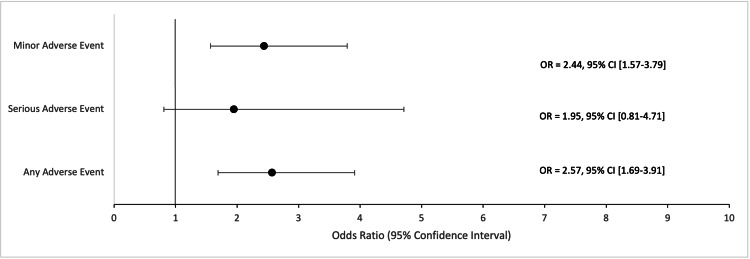
Propensity score matched multivariate odds ratios in COVID-19 (+) patients who underwent humeral fracture surgery. Forest plot displaying multivariate odds ratios and corresponding 95% confidence intervals for adverse events in COVID-19-positive patients who underwent humerus fracture surgery. The comparison group in the logistic regression model was the 1:10 propensity score matched COVID-19-negative cohort. This model controlled for age, sex, and comorbidities. OR = Odds Ratio; CI = Confidence Interval

Multivariate analysis using a logistic regression controlled for patient demographics and comorbidities was performed on the aggregated adverse event categories (Table 6). In the unmatched comparison, COVID-19-positive patients had higher odds of any adverse events (2.25; 95% CI: 1.56-3.26; p < 0.001) and minor adverse events (2.1; 95% CI: 1.43-3.07; p < 0.001) (Figure 1). In the matched comparison, statistical significance was maintained where COVID-19-positive patients had higher odds of any adverse events (2.57; 95% CI: 1.69-3.91; p < 0.001) and minor adverse events (2.44; 95% CI: 1.57-3.79; p < 0.001) (Figure 2).

**Table 6:** Multivariate analysis of adverse events by COVID-19 status. *1:10 matching of year of birth, sex, and all comorbidities OR = Odds Ratio; CI = Confidence Interval

	Multivariate Odds Ratios					
	Propensity Score Matched Odds Ratio*			Unmatched Odds Ratio		
	OR	95% CI	p-value	OR	95% CI	p-value
Any Adverse Event	2.57	1.69-3.91	<0.001	2.25	1.56-3.26	<0.001
Serious Adverse Event	1.95	0.81-4.71	0.090229	1.96	0.97-3.93	0.05955
Minor Adverse Event	2.44	1.57-3.79	<0.001	2.1	1.43-3.07	<0.001

## Discussion

The COVID-19 pandemic significantly impacted orthopedics from 2020 to 2022. This has resulted in a call for literature that can assist orthopedic surgeons in the management of patients during potential future pandemics [2]. To date, quality studies have investigated adverse events of COVID-19-positivepatients undergoing surgical treatment for lower extremity fractures [15,16]. Similar investigations in the surgical treatment of upper extremity fractures have not been completed, except for a few institutional studies [17]. To our knowledge, this is the first study with high statistical power that addresses adverse events in COVID-19-positive patients who underwent surgery for humerus fractures.

This study demonstrated that COVID-19-positive patients were more likely to experience adverse events after surgery for humerus fractures. When comparing all COVID-19-negative and COVID-19-positive patients, COVID-19-positive patients had significantly higher odds of experiencing any adverse events and minor adverse events. The propensity score matching of the COVID-19-negative cohort to the COVID-19-positive cohort allowed us to control for the size difference in the cohorts, patient characteristics, and patient comorbidities. After propensity score matching, the significantly higher odds of experiencing any adverse events and minor adverse events in the COVID-19-positive cohort held true. This is consistent with the findings from a prior study that examined adverse events in COVID-19-positive patients undergoing surgical fixation of lower extremity fractures [15,16]. Interestingly, those two studies also found that COVID-19-positive patients demonstrated significantly higher odds of experiencing serious adverse events as well, which was not observed in this present study.

Univariate analysis of the matched COVID-19-negative cohort and the COVID-19-positive cohort found that COVID-19 status had no direct effect on developing cardiac arrest, pancreatitis, pulmonary embolism, sepsis, urinary tract infection, wound dehiscence, and pneumonia after humerus fracture surgery. However, it was found that myocardial infarction and acute kidney injury occurred more frequently in the COVID-19-positive cohort when compared to the matched COVID-19-negative cohort. These associations are compelling due to related findings outlining the pathophysiologic mechanisms of COVID-19 on host tissues. The virus' ability to directly damage myocardial cells, invoke inflammation, and destabilize coronary plaques is theorized to contribute to the development of postoperative myocardial infarction in COVID-19-positive patients [18,19]. This explains why COVID-19-positive patients in this study were more likely to experience a myocardial infarction. It also can damage endothelial cells and renal tubules and promote the release of local inflammatory mediators [20], all of which contribute to the development of or progression of acute kidney injury. Furthermore, hospitalized COVID-19-positive patients with an active acute kidney injury have been found to have increased rates of admission to the intensive care unit and in-hospital mortality [21].

These findings suggest that orthopedic surgeons should be aware of the increased risk for cardiorenal complications in COVID-19-positive patients. Cardiovascular evaluation is used to stratify the cardiac risk of patients so that orthopedic surgeons can make appropriate clinical decisions to mitigate adverse cardiovascular events [22]. A future implication of this study is to use COVID-19 infection as a predictor to consider when dictating management of cardiovascular risk in patients undergoing surgical fixation of humerus fractures. Further investigation with high-power studies that examine the role that COVID-19 infection has on the development of cardiovascular events following upper extremity orthopedic procedures is needed.

The univariate analysis also found that there was a significant difference in the rate of surgical site infection experienced by the COVID-19-positive cohort when compared to the matched COVID-19-negative cohort. Statistical analysis using Pearson's chi-squared test with Yates continuity correction determined that there was a statistically significant (p < 0.001) difference for surgical site infection between the zero patients in the 1,320 matched COVID-19-negative sample and the zero patients in the 132 COVID-19-positive sample. This unusual finding could be attributed to the probability of committing a type 1 error where the null hypothesis is falsely rejected when it is true. In this study, steps taken to reduce type 1 error included using an appropriate significance level (p < 0.05), creating a 1:10 propensity score matched COVID-19-negative control, and re-replication of the results. Another explanation of this finding involves a limitation of this study relating to the identification of patient diagnoses using ICD-10 codes. Given that few surgical site infections were found in the unmatched and matched COVID-19-negative cohorts, there is a chance that the ICD-10 codes used in this study did not adequately query for surgical site infections. Future studies on this topic that utilize the Change Healthcare dataset should critically assess all potential ICD-10 codes that could be used to identify a surgical site infection diagnosis.

The results of this study are subject to several additional limitations. As mentioned, it is possible that all humerus fracture patients who were COVID-19-positive were not identified using these codes. We were also unable to determine the date at which ICD-10 codes were assigned to patients for adverse events. Therefore, an accurate range in which postoperative events were diagnosed could not be reported. While significant and of interest, these limitations are consistent with similar large database studies examining these effects over the last couple of years.

A final important limitation of our study is that it examines all types of humerus fractures undergoing surgical fixation. To obtain enough patients to conduct a statistical analysis representing the general population, it was imperative to include all types of humerus fractures. Including all types of humerus fractures in this study did not allow us to control for variables that could impact the rate of adverse events that may be unique to specific humerus fractures. Fracture location, classification, and individual patient characteristics determine the modality of fixation which in turn can influence humerus fracture surgery outcomes. A future direction of this study is to conduct a subgroup analysis on how COVID-19 infection impacts the risk for adverse events in specific fracture types and operative techniques.

## Conclusions

In conclusion, patients who test positive for COVID-19 are more likely to face adverse events and have worse clinical outcomes when undergoing humerus fracture surgery. Orthopedic surgeons should use these data to help guide clinical decision-making regarding patients who are undergoing humerus fracture fixation with an accompanying diagnosis of COVID-19.
